# Cystic echinococcosis in northern Tanzania: a pilot study in Maasai livestock-keeping communities

**DOI:** 10.1186/s13071-022-05518-x

**Published:** 2022-10-28

**Authors:** Francesca Tamarozzi, Tito Kibona, William A. de Glanville, Tauta Mappi, Elly Adonikamu, Anande Salewi, Kennedy Misso, Venance Maro, Adriano Casulli, Azzurra Santoro, Federica Santolamazza, Blandina T. Mmbaga, Sarah Cleaveland

**Affiliations:** 1 IRCCS Sacro Cuore Don Calabria Hospital, Negrar di Valpolicella, Verona Italy; 2 Kilimanjaro Clinical Research Institute, Moshi, Tanzania; 3 University of Glasgow, Glasgow, UK; 4 Kilimanjaro Christian Medical Centre, Moshi, Tanzania; 5 Kilimanjaro Christian Medical University College, Moshi, Tanzania; 6 Istituto Superiore di Sanità, Rome, Italy

**Keywords:** *Echinococcus granulosus sensu lato*, Cystic echinococcosis, Maasai, Northern Tanzania, Prevalence, Genotypes, Human, Livestock, Ultrasound

## Abstract

**Background:**

There are close similarities between the life-cycles of *Echinococcus granulosus sensu lato* (*E. granulosus s.l.*) that causes cystic echinococcosis (CE) in humans and *Taenia multiceps/Coenurus cerebralis* that causes cerebral coenurosis in small ruminants. Recent evidence highlights that livestock in Maasai communities of northern Tanzania are suffering from increases in the prevalence of cerebral coenurosis, leading to concerns about a possible concurrent increased risk of human CE. The aim of this study was to estimate the prevalence of human abdominal CE and the prevalence and species/genotypes of* E. granulosus s.l.* in livestock in Maasai communities.

**Methods:**

Human CE was diagnosed by abdominal ultrasound on volunteers aged ≥ 7 years in five villages in the Longido and Ngorongoro Districts in northern Tanzania. Infection in ruminants was evaluated through inspection in local abattoirs, followed by molecular identification of one cyst per animal, with a priority for hepatic cysts, using PCR targeting of the cytochrome* c* oxidase I gene (*COX1*), followed by restriction fragment length polymorphism and multiplex PCR, and sequencing of non-*E. granulosus s.l.* samples.

**Results:**

Ultrasound was performed on 823 volunteers (*n* = 352 in two villages in Longido District, and* n* = 471 in three villages of Ngorongoro). Hepatic CE cases were diagnosed only in Ngorongoro (*n* = 6; 1.3%), of which three had active cysts. Village-level prevalence of CE ranged between 0 and 2.4%. Of the 697 ruminants inspected, 34.4% had parasitic cysts. Molecular identification was achieved for 140 of the 219 (63.9%) cysts sampled. *E. granulosus s.l*. and *T. hydatigena/Cysticercus tenuicollis *were identified in 51.4% and 48.6%, respectively, of livestock cysts. *E. granulosus s.l.* was identified in livestock from both Longido (35.3% of 116 genotyped cysts) and Ngorongoro (91.2% of 34 genotyped cysts). Of the total of 72 *E*. *granuslosus s.l.* cysts identified in livestock, 87.5% were *E. granulosus sensu stricto* (G1–G3 genotypes), 9.7% were *E. ortleppi* (G5) and one cyst was *E. canadensis* (G6–10). The three active human cysts, which were removed surgically, were G1–G3 genotypes.

**Conclusions:**

Multiple species/genotypes of *E. granulosus s.l.* are circulating in Maasai communities of northern Tanzania. Human CE was detected in villages of Ngorongoro District and a high prevalence of echinococcal cysts was observed in livestock in both districts. More precise estimation of the prevalence in this area and a better understanding of the specific risk factors for CE among Maasai communities in northern Tanzania is needed. Interventions targeting transmission routes common to both *E. granulosus s.l.* and *T. multiceps* would have dual benefits for preventing both human and livestock disease.

**Graphical Abstract:**

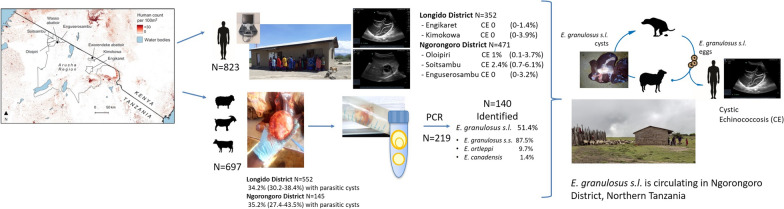

## Background

Cystic echinococcosis (CE) is a chronic, neglected zoonosis which WHO includes in its list of priority neglected tropical diseases [1]. The disease is caused by infection with the larval stage of the tapeworm *Echinococcus granulosus sensu lato* (*E. granulosus s.l.*) [2]. The life-cycle of this parasite involves canids as definitive hosts, which harbour the adult tapeworms in the intestine, and livestock as intermediate hosts where the larval stage develops as fluid-filled cysts in internal organs [3]. The infection is particularly prevalent in extensive and semi-nomadic livestock-keeping communities in Asia, Africa, South America, Australia and parts of Europe [4] and is linked with poor living conditions [5]. People become infected through the ingestion of eggs shed through dog faeces. In successful infections, echinococcal cysts develop most often in the liver [6].

CE cysts evolve through and can present in different stages, from active (biologically viable) to inactive (biologically quiescent or not viable) stages [7, 8]. Infected individuals may remain asymptomatic for many years (or even life-long) until cyst growth triggers clinical signs, with possible severe, even fatal, complications. These include compression on neighbouring structures/organs and rupture of the cyst into hollow structures (e.g. bronchi, biliary ducts) or body cavities (e.g. peritoneal or pleural cavity) with consequent dissemination, allergic reactions and bacterial superinfection [6]. The clinical management of human CE is complex, with active cysts requiring long-term treatment with antiparasitic drugs or invasive procedures such as surgery [9]. Furthermore, patients require years-long follow-up to promptly detect recurrence or reactivation [9].

CE has a high veterinary and human public health impact on endemic communities. An estimated 1.2 million people are infected with CE worldwide, with medical and veterinary losses estimated at about 3 billion USD/year as a result of costs relating to diagnosis and treatment of human cases and livestock production losses [10]. CE is a zoonosis prioritized for action by the WHO in the 2012–2020 and 2021–2030 roadmaps on Neglected Tropical Diseases [1, 11], with a recommendation to “map disease prevalence to establish baseline data” highlighted among the critical actions required towards infection control [1]. However, the epidemiological situation in many areas of the world is still extremely scant, particularly in sub-Saharan Africa [4, 12], despite the large population of pastoralists (estimated at 268 million [13]) who are likely to be represent a high-risk population for CE.

In pastoral communities of Ngorongoro District, northern Tanzania, a prevalence of *E. granulosus s.l.* infection of 63% was reported in sheep between 1998 and 2001 based on the results of abattoir surveys [14], and a surgical incidence of 10 cases per 100,000 general population per year was reported between 1990 and 2003 through an analysis of hospital records [14]. These figures compare with those of hyper-endemic communities worldwide [4] and are much higher than the annual incidence of 1–5 per 100,000 general population indicated by the WHO as the threshold figure for “high endemicity” [15]. In addition, clinical incidence does not reflect the real epidemiologic situation since the majority of human infections, irrespective of their clinical severity, remain undetected [16]. Community-based ultrasound (US) surveys are needed to obtain a more accurate epidemiological picture of human CE, and to assess the need for interventions [16]. However, no population-based US surveys have been carried out in these communities since the late 1980s, when Macpherson and colleagues [17, 18] reported an abdominal CE prevalence of 1% in Maasai pastoralists of northern Tanzania.

A further rationale for generating accurate contemporary CE data in pastoral communities in northern Tanzania relates to the extremely high prevalence of another cestode parasite, *Taenia multiceps*, that has been detected as the cause of a fatal disease (cerebral coenurosis) in small ruminants in these communities [19, 20]. This parasite, which has a similar life-cycle to *E. granulosus*, involves domestic dogs as definitive hosts and small ruminants as intermediate hosts. It has recently been shown to affect > 90% of flocks in pastoral communities of northern Tanzania, with 11–34% dying of the disease each year [19]. This high prevalence is reflected in very high levels of concern of pastoralists about the disease [21]. Rare cases of zoonotic transmission have also been reported, with human cases reported to mimic hydatid disease [22–24]. The reasons for the very high current levels of coenurosis in pastoral communities are not well understood, but may reflect changes in livestock ownership (with evidence for the shift from keeping cattle to keeping sheep and goats [25]), management practices (such as an increase in supplementary feeding of livestock) and the expansion of dog populations [26]. As a result of the similarity in life-cycles and transmission routes, the same factors driving the high prevalence of *T. multiceps* may also be driving an increase in the transmission of *E. granulosus s.l,* which, in people, might become evident only in the years to come due to the long latency between infection and clinical manifestations in humans.

This study, to be considered as a pilot exploratory study, therefore specifically aimed to estimate the current prevalence of human abdominal CE and prevalence and species/genotypes of *E. granulosus s.l.* in livestock in a selection of Maasai communities, with the overall aim to provide baseline epidemiological data to support public health decision-making.

## Methods

Ethics approval was granted by the Kilimanjaro Christian Medical University College, Moshi, Tanzania (09/08/2019), the National Health Ethics Review Committee, National Institute for Medical Research, Dar es Salaam, Tanzania (NIMR/HQ/R.8c/Vol.I/732), the University of Glasgow College of Medical, Veterinary and Life Sciences Ethics Committee (no. 200180193) and the School for Veterinary Medicine Research Ethics Committee (EA36/20).

### Prevalence of abdominal cystic echinococcosis in humans

**Fig. 1:**
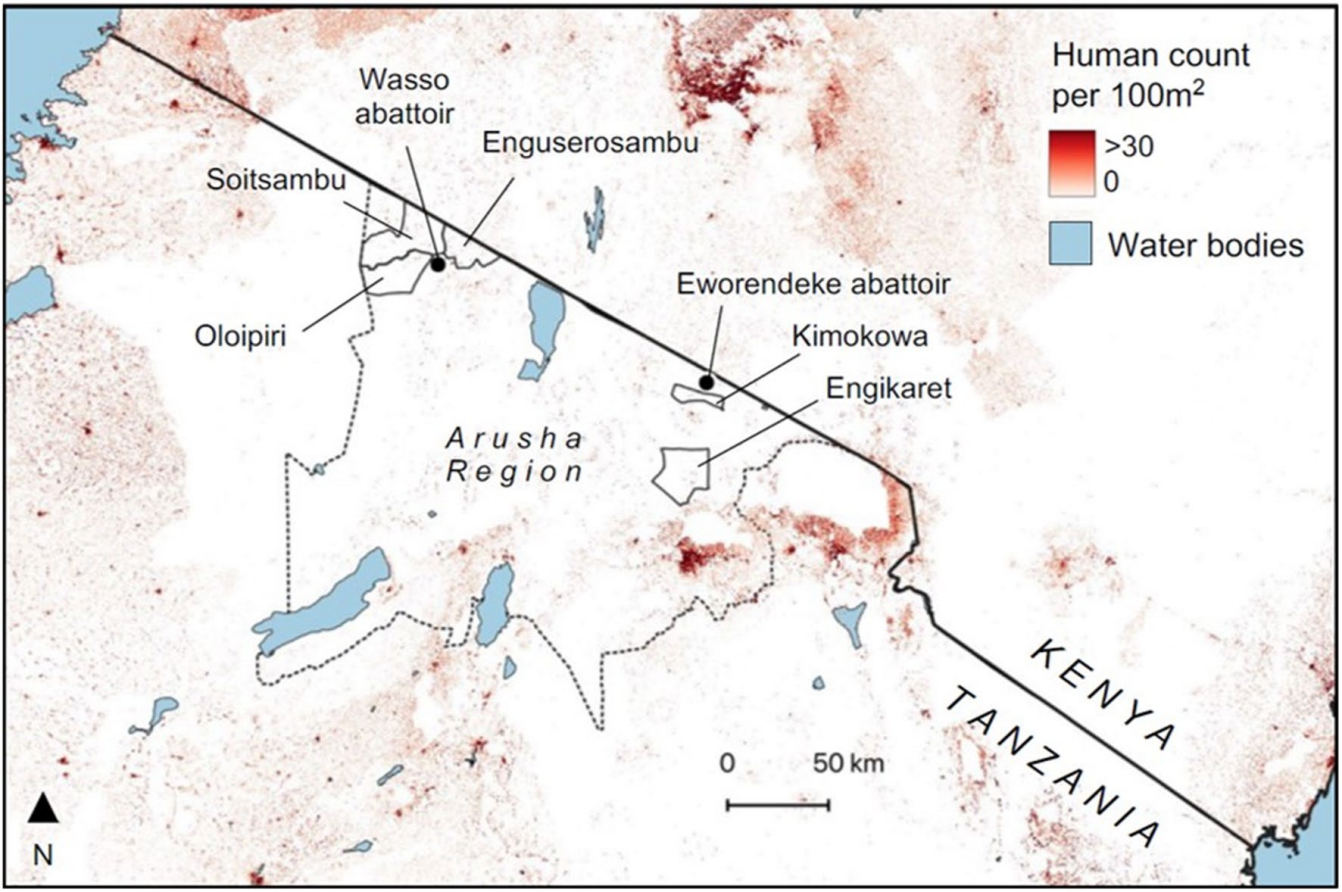
Map showing the locations of five villages in which ultrasound surveys were conducted on volunteers and the locations of two abattoirs where livestock sampling was conducted, in Ngorongoro District and Longido District, Arusha Region, northern Tanzania. The map was created using QGIS version 3.22.1. Boundary shapefiles are from https://gadm.org/ and http://geoportal.icpac.net. Human population counts for 2020 at a resolution of 3 arc seconds (approximately 100 m at the equator) from https://hub.worldpop.org/

A cross-sectional US survey was carried out in November 2019 in five predominantly Masaai pastoral villages of Ngorongoro and Longido Districts of the Arusha Region, northern Tanzania (Fig. 1).

Villages were selected from those that had participated in an earlier zoonoses research programme with initial selection based on a generalized random tessellation stratified sampling approach (additional details described in [27]). Villages from Ngorongoro and Longido Districts were selected for this study on the basis of their agro-ecological characteristics, their predominantly pastoral households and their proximity to district abattoirs in order to allow human and livestock prevalence data to be linked. The human population size of these communities in 2019 was based on projections from the 2012 census (Tanzania National Bureau of Statistics, Dodoma, Tanzania; https://www.nbs.go.tz/index.php/en/), assuming a growth rate of 2.9% [28]. After gaining consent from community leaders, meetings in each village were organized to explain in both Kiswahili and Maasai languages the aim and protocol of the project, to provide information on CE and cerebral coenurosis and to invite potential participants to attend an ultrasound (US) screening session, the site and date of which were communicated in advance. Participants were enrolled after they presented spontaneously to the study venues, on a first-come first-served basis. Participants of both sexes were eligible if aged ≥ 7 years and living in the target area. The threshold age was decided upon based on the practicalities of carrying out surgery for CE within the project context. Written/fingerprinted informed consent/assent was obtained from individuals, together with those of parents/guardians of minors, willing to participate in the survey. Participation was voluntary and refreshments were offered to compensate for travelling to the study sites.

Five villages were selected based on available time and resources. Given the small number of villages included, our aim was to detect CE cases and derive estimates of prevalence at the village-level rather than across the region as a whole. A sample of 230 people in each village would have allowed us to rule out a prevalence of CE of ≥ 1% with 90% confidence if no cases were detected.

Each surveyed participant was registered on a case report form, uniquely identified by an anonymous alphanumeric code that was used on all study documents and data files. Abdominal US was carried out in a confidential environment (i.e. a room or building separate from the waiting and enrolment place), using a portable SonoSite M-Turbo machine (Fujifilm, Seattle, WA, USA) equipped with a convex probe, by a physician sonographer (FT) with extensive experience in the diagnosis of CE or by two study physicians (EA, ASal, who were trained in point-of-care abdominal ultrasonography during the study) under constant direct supervision of the first sonographer. In participants with abdominal CE, defined as a focal lesion with stage-specific pathognomonic signs of parasitic aetiology on US, data were recorded on cyst localization, number, size and stage according to the WHO Informal Working Group on Echinococcosis (WHO-IWGE) classification [9]. Briefly, CE1 (unilocular fluid-filled cyst with double-wall), CE2 (fluid-filled cysts with daughter cysts), CE3a (unilocular fluid-filled cysts with detached parasitic layers) and CE3b (daughter cysts in a solid matrix with folded hypoechoic parasitic layers) cysts were classified as active, and CE4 (solid content with folded hypoechoic parasitic layers) and CE5 (CE4 with evident egg-shell calcifications) cysts were classified as inactive. Individuals with CE were managed within the project framework according to the WHO-IWGE Expert Consensus recommendations [9]. In the case of suspected CE lesions (defined as focal lesions without sonographic pathognomonic signs of parasitic or non-parasitic origin), a commercial immunochromatographic test (VIRapid Hydatidosis; Vircell, Granada, Spain) would be performed. Advanced imaging (e.g. contrast-enhanced computed tomography scan) was also available at the Kilimanjaro Christian Medical Centre (Moshi, Tanzania) if required. If lesions other than CE with clear or potential medical relevance were detected, the participant was provided a written report and advised to attend an appropriate health facility at their own cost.

### Cystic echinococcosis in slaughtered livestock

A survey was carried out in district authority abattoirs, located in Wasso village in Ngorongoro District and in Eworendeke village in Longido District, to determine the prevalence of *E. granulosus s.l.* infection in cattle, sheep and goats. These abattoirs serve livestock-keeping communities in Ngorongoro and Longido districts and receive livestock from across each district, including the areas where the human US screening was conducted.

On the expectation that around 50% of animals would have detectable parasitic cysts and that 50% of these cysts would be due to *E. granulosuis s.l.* [14, 21], our target sample size in each slaughterhouse to estimate a 25% prevalence of *E. granulosus s.l.* among livestock with 5% error and 95% confidence interval was 290 animals, with a target of around 100 animals of each species.

**Fig. 2:**
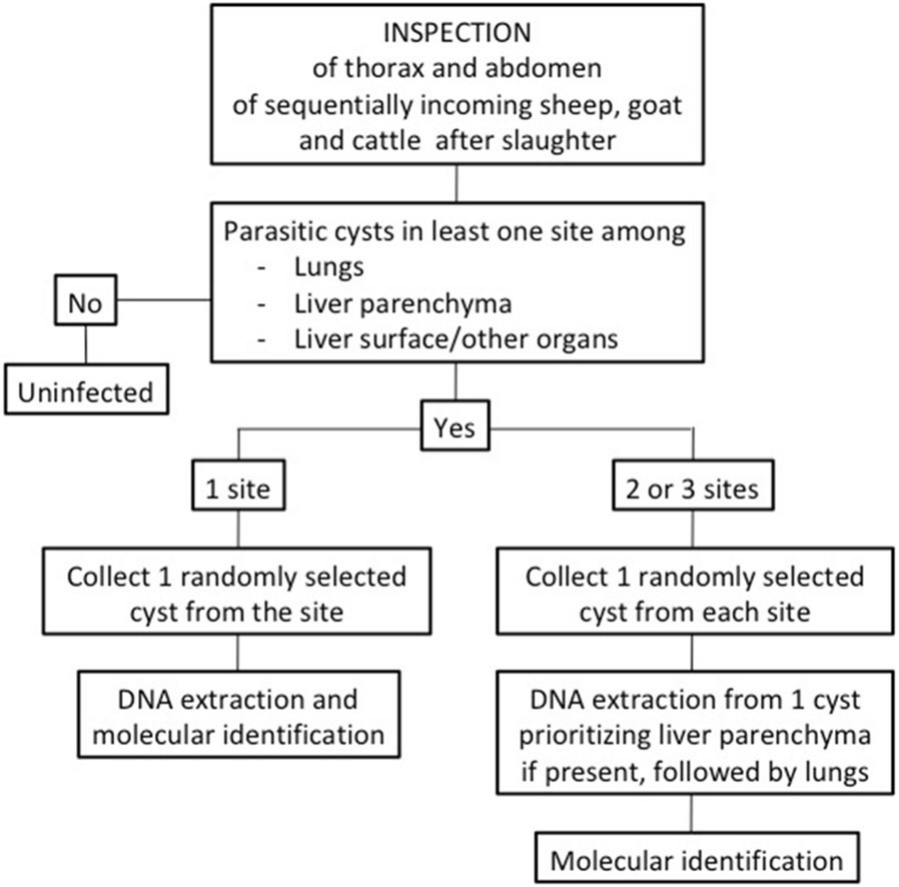
Flow diagram showing livestock inspection at abattoirs and the sampling strategy of parasitic cysts

All consecutive slaughtered ruminants accessing the abattoirs between October and December 2020 were assigned a unique code and were inspected by local abattoir inspectors for the presence of visible cysts in the abdomen and lungs. If present, one random cyst was removed from each of three sites, namely the liver parenchima, the liver surface/peritoneum and the lungs (Fig. 2), and preserved in 70% ethanol. Species, age group based on pairs of adult incisors and localization of visible cysts were reported on a case report form.

Before the start of the sampling period, training was provided to the inspectors on the study procedures and identification of parasitic cysts in the form of on-line sessions due to COVID-19 restrictions. However, no tentative identification of parasitic cysts at the genus level (*Echinococcus* or *Cysticercus*) was requested the inspectors since the training that could be provided was not considered sufficient to guarantee correct identification. Due to COVID-19-related disruptions to travel, in-person morphological characterization of cysts by experienced study staff was not possible. Attempts were made to use video-conferencing to differentiate ethanol-preserved specimens of *E. granulosus s.l.* and *Taenia hydatigena/Cysticercus tenuicollis*, another tapeworm transmitted between canids and livestock forming cyst-like metacestodes typically hanging from the surface of viscera. However, this was not successful, and identification therefore relied on molecular analysis of a subset of removed cysts, selected as detailed in section Molecular analysis of metacestodes.

### Molecular analysis of metacestodes

DNA was extracted from one parasitic cyst per animal. In animals with cysts removed from more than one localization, DNA extraction was prioritized from the cyst removed from the hepatic parenchyma, followed by lungs and finally liver surface/peritoneum. This scheme was used since *E. granulosus s.l*. cysts more frequently affect the hepatic parenchyma, followed by the lungs, while *C. tenuicollis* is most often localized on serous surfaces. The DNA was extracted in the laboratory of the Kilimanjaro Clinical Research Institute (KCRI) from a 1.5- to 2-cm fragment of each cyst wall using the DNeasy Blood and Tissue Kit (Qiagen, Hilden, Germany) following the manufacturer’s instructions. Nuclease-free water was included as a negative control in each working session to verify absence of contamination. The DNA was shipped to the WHO Collaborating Centre on the Epidemiology, Detection and Control of Cystic and Alveolar Echinococcosis (Istituto Superiore di Sanità, Rome, Italy). The molecular identification of the metacestode species was carried out following the protocol published by Santolamazza et al. [29]. Briefly, a fragment of the mitochondrial cytochrome Oxidase I gene (*COX1*) was amplified by PCR and the 444-bp fragment digested by *Alu*I. Restriction fragment length polymorphism (RFLP) identifies G1–G3 genotypes (*Echinococcus granulosus *sensu stricto [*E.granulosus s.s.*]) from G4 to G10 *E. granulosus* genotypes; the latter are further identified into G4, G5, G6/7 and G8/10 by band patterns after multiplex PCR. Non-*E. granulosus s.l.* samples were submitted for bidirectional Sanger sequencing of the* COX1* PCR product for species identification. Resulting forward and reverse sequences were aligned to generate single consensus sequences, which were compared with nucleotide sequences deposited in GenBank using BLASTn (nucleotide Basic Local Alignment Search Tool).

### Data analysis

Continuous variables were described as mean and standard deviation or median and interquartile range, as appropriate; categorical variables were described as numbers and percentages. Prevalence of human CE at the village-level was reported with exact 95% binomial confidence intervals (CIs). Given the small number of villages in each district, we did not derive a district-level 95% CI or directly compare district-level prevalence. We derived 95% CIs for abattoir-level prevalence (since they receive animals from a wide area) and compared abattoir-level prevalence using a Chi-square (*χ*) test. Exact binomial confidence intervals were derived in R version 4.1.1. using the *binom* package (R Foundation for Statistical Computing, Vienna, Austria). The Chi-square test was performed using base functions in R.

## Results

### Prevalence of abdominal CE in humans

**Fig. 3:**
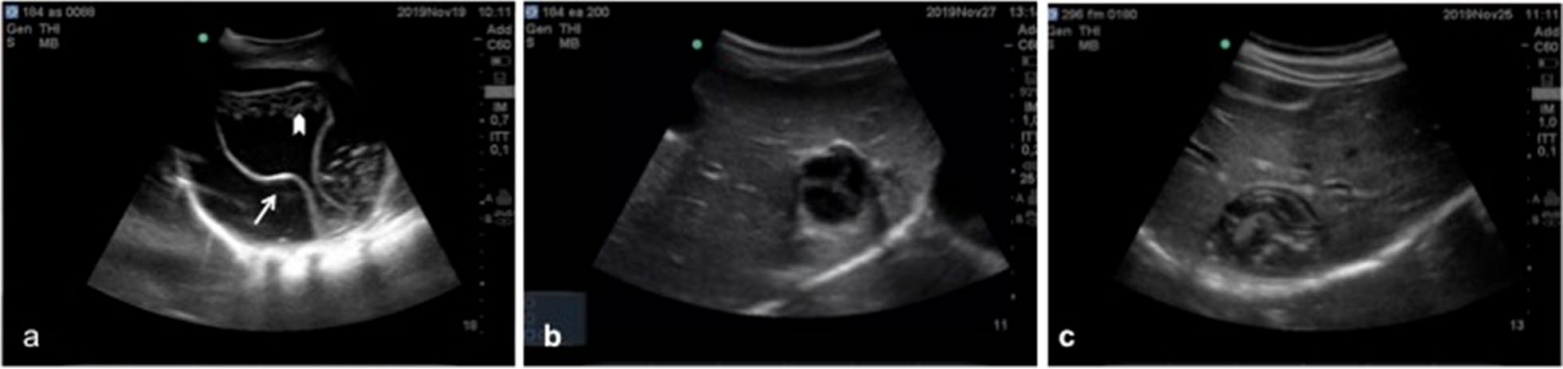
Example images of active (**a**,** b**) and inactive (**c**) echinococcal cysts diagnosed by ultrasound in communities of Ngorongoro. **a** 13-cm CE3a cyst showing detached parasitic membranes (arrow) but also small daughter cysts (arrowhead). **b** 4.5-cm CE2 cysts characterized by daughter cysts filling the entire cyst. **c** 5.6-cm CE4 cyst characterized by the “ball of wool” appearance of the folded parasitic membranes in the pseudo-solid matrix filling the cyst cavity. See section Prevalence of abdominal cystic echinococcosis in humans for description of cyst stages

Abdominal US was carried out on 823 volunteers, 507 (61.60%) females and 316 (38.4%) males, in five Maasai pastoral communities of two districts (Longido, 2 villages, *n* = 352 people and Nogorongoro, 3 villages, *n* = 471 people) of northern Tanzania (Table 1), comprising 2.6% of the estimated population ≥ 5 years of age in the study villages of these areas. In Longido District, no abdominal CE was diagnosed, and no participant reported any previous treatment for CE. In Ngorongoro District, six abdominal CE cases were diagnosed The village-level prevalence in the study districts ranged between 0 and 2.4% (Table 1). All CE cysts were single and localized in the liver; three were in inactive stages (*n* = 2 CE4; *n* = 1 CE5) and three were active (Fig. 3): one cyst was in CE2 stage; two large cysts (> 10 cm) were in CE2/CE3a stage (one of which symptomatic with abdominal pain and lack of appetite). These latter two cysts had a peculiar morphology showing both detached parasitic layers and small daughter cysts. Individuals with inactive CE were aged 30, 60 and 79 years while individuals with active CE were aged 7, 18 (siblings of the same household) and 30 years. No participant reported having received specific treatment for CE in the past. The three participants with active CE cysts were treated surgically with adjunct prophylaxis with albendazole in the Kilimanjaro Christian Medical Centre, (Moshi, Tanzania) at the project’s expense and made a full clinical recovery.

**Table 1:** Results of ultrasound screening for abdominal cystic echinococcosis

District	Community	No. of individuals having US	No. of females having US (%)	Age (years( [SD; range]	No. of individuals with CE	Prevalence of CE (95% CI)	Details of CE cases^a^
Longido	Engikaret	260	191 (73.5%)	37 [22; 7–96])	0	0 (0–1.4%)	–
	Kimokowa	92	58 (63.0%)	43 [21; 7–95]	0	0 (0–3.9%)	–
	*Total*	*352*	*249 (70.7%)*	*39 [21; 7–96]*	*0*	*0*	
Ngorongoro	Oloipiri	191	108 (56.5%)	37 [24; 7–99]	2	1.0% (0.1–3.7%)	- 12-cm cyst at CE3a stage in 7-year-old boy - 4.5-cm cyst at CE2 stage in 18-year-old boy
	Soitsambu	166	81 (48.8%)	38 [20; 7–87]	4	2.4% (0.7–6.1%)	- 10-cm cyst at CE3a stage in 30-year-old woman - 5-cm cyst at CE4 stage in 30-year-old man - 4-cm cyst at CE5 stage in 79-year-old woman - 4-cm cyst at CE4 stage in 60-year-old woman
	Enguserosambu	114	69 (60.5%)	33 [23; 7–91]	0	0 (0–3.2%)	–
	*Total*	*471*	*258 (54.8%)*	*37 [22; 7–99]*	*6*	*1.3%*	

* CE* cystic echinococcosis,* CI* confidence interval,* SD* standard deviation,* US* ultrasound

^a^Cyst stages are described in section Prevalence of abdominal cystic echinococcosis in humans

Other findings observed on US that had clinical relevance (i.e. excluding simple cysts and hepatic typical hemangiomas) were prostate hypertrophy (*n* = 19), hepatosteatosis (*n* = 18), gallstones (*n* = 5), uterine myofibroma (*n* = 4), signs of urinary tract infection (*n* = 3), focal liver lesions suggestive of neoplasm (*n* = 2), bladder neoplasm (*n* = 2), cirrhosis (*n* = 2), complex ovarian cysts (*n* = 2) and polycystic kidney disease (*n* = 1).

### Parasitic cysts in slaughtered ruminants

A total of 697 slaughtered ruminants were inspected in the two abattoirs. Of these, 552 animals were from the abattoir in Longido District (105 cattle, 206 sheep, 241 goats) and 145 were from the abattoir in Ngorongoro District (113 cattle, 6 sheep, 26 goats). Visible abdominal and/or thoracic parasitic cysts were observed in 240 of the 697 (34.4%) animals: 189 of the 552 animals inspected in Longido (34.2%; 95% CI 30.2–38.4) and 51 of the 113 inspected animals in Ngorongoro (35.2%; 95% CI 27.4–43.5). There was no evidence of a difference in cyst prevalence between the districts (*χ*^2^ = 0.01, *P* = 0.9). The prevalence of cysts increased slightly with the age of animals, from 29.5% (28/95) in animals with no adult incisors (aged < 12–18 months) to 35.6% (85/239) in animals with four pairs of adult incisors (42–48 months of age). A total of 247 cysts (18 from liver parenchyma, 94 from lungs, 135 from peritoneum/liver surface) were collected from 236 animals, as detailed in the Methods section and in Fig. 2, and shipped to KCRI in ethanol for analysis. Details of the inspected animals and parasitic cysts are shown in Table 2.

**Table 2:** Results of the evaluation of parasitic cysts in slaughtered ruminants

District	Livestock	No. of animals inspected	No. of animals with cysts (% of inspected)	No. of cysts collected^a^	No. of cysts from which DNA was extracted^b^	No. of species identified/successfully genotyped (%)	
						*Echinococcus granulosus*	*Echinococcus hydatigena*
Longido	Bovine	105	42 (40.0%)	Total = 42 Liver = 0 Lung = 41 Perit. = 1	Total = 41 Liver = 0 Lung = 41 Perit. = 0	Total = 31/41 (75.6%) Liver = 0 Lung = 31 [27 Egss, 3 Eo, 1 Ec] Perit. = 0	Total = 0/41 (0.00%) Liver = 0 Lung = 0 Perit. = 0
	Ovine	206	21 (10.2%)	Total = 21 Liver = 0 Lung = 11 Perit. = 10	Total = 15 Liver = 0 Lung = 6 Perit. = 9	Total = 8/12 (66.7%) Liver = 0 Lung = 4 [4 Egss] Perit. = 4 [2 Egss, 1 Eo, 1 Egsl]	Total = 4/12 (33.3%) Liver = 0 Lung = 1 Perit. = 3
	Caprine	241	126 (52.3%)	Total = 124 Liver = 3 Lung = 6 Perit. = 115	Total = 121 Liver = 3 Lung = 6 Perit. = 112	Total = 2/63 (3.2%) Liver = 0 Lung = 1 [Eo] Perit. = 1 [Eo]	Total = 61/63 (96.8%) Liver = 0 Lung = 1 Perit. = 60
	*Total*	*552*	*189 (34.2%)*	*187*	*177 (94.6%)*	*41/116 (35.3%)*	*65/116 (56.0%)*
Ngorongoro	Bovine	113	40 (35.4%)	Total = 49 Liver = 14 Lung = 31 Perit. = 4	Total = 35 Liver = 14 Lung = 19 Perit. = 2	Total = 28/28 (100.0%) Liver = 10 [10 Egss] Lung = 16 [15 Egss, 1 Eo] Perit. = 2 [2 Egss]	Total = 0/28 (0.0%) Liver = 0 Lung = 0 Perit. = 0
	Ovine	6	1 (16.7%)	Total = 1 Liver = 0 Lung = 1 Perit. = 0	Total = 1 Liver = 0 Lung = 1 Perit. = 0	Total = 1/1 (100%) Liver = 0 Lung = 1 [Egss] Perit. = 0	Total = 0/1 (0.00%) Liver = 0 Lung = 0 Perit. = 0
	Caprine	26	10 (30.8%)	Total = 10 Liver = 1 Lung = 4 Perit. = 5	Total = 6 Liver = 1 Lung = 1 Perit. = 4	Total = 2/5 (40.0%) Liver = 1 [Egss] Lung = 0 Perit. = 1 [Egss]	Total = 3/5 (60.0%) Liver = 0 Lung = 1 Perit. = 2
	*Total*	*145*	*51 (35.2%)*	*60*	*42 (70%)*	*31/34 (91.2%)*	*3/34 (8.8%)*

*Ec Echinococcus canadensis* (G6–10 genotypes),* Egsl*  *Echinococcus granulosus sensu lato* [further genotyping was not achieved],* Egss Echinococcus granulosus sensu stricto* (G1–G3 genotypes),* Eo* *Echinococcus ortleppi* (G5 genotype),* Perit.* peritoneal cysts on organ surface

^a^One random cyst was collected from each infected site among liver parenchyma, lungs and peritoneum

^b^DNA was extracted from one cyst per animal. For animals with > 1 cyst collected, priority was given to the cyst collected from the liver parenchyma, followed by liver and lastly from the peritoneum

### Molecular identification of metacestodes

The three cysts surgically removed from the individuals with active CE cysts were identified as *E. granulosus s.s. * (G1–G3 genotypes)*.*

DNA was extracted from 219 cysts sampled from slaughtered ruminants (76 from cattle, 16 from sheep, 127 from goats), one cyst per animal, as detailed in the Methods section and in Fig. 2. Identification was achieved for 140 (63.9%) cysts, while no amplification was obtained from 79 (36.1%) cysts. Results of the typing of cysts in livestock are detailed in Table 2. *Echinococcus granulosus s.l*. was identified in 72 cysts (51.4% of genotyped cysts) from both districts, but the proportion of *E. granulosus s.l.* cysts among genotyped cysts was significantly higher in Ngorongoro District (*n* = 31/34, 91.2%) than in Longido District (*n* = 41/116, 38.7%) (*χ*^2^ = 32.8, *P* ≤ 0.0001). The remaining genotyped cysts (*n* = 68, 48.6%) were identified as *T. hydatigena/C. tenuicollis*.

Of the *E. granulosus s.l.* cysts, *E. granulosus s.s.* was identified in 63/72 (87.5%) of cases; specifically, in 91.5% of echinococcal cysts from cattle, 77.8% of echinococcal cysts from sheep and 50% of echinococcal cysts from goats. *Echinococcus ortleppi* (*E. granulosus s.l.* G5 genotype) was identified in 9.7% of echinococcal cysts, in all three livestock species and in both districts. *Echinococcus canadensis* (*E. granulosus s.l*. G6-10 genotypes) was identified in one cyst from one bovine in Longido, while in one case *E. granulosus s.l.* identification at species/genotype level was not possible.

## Discussion

East Africa is considered to be among the areas of the world with the highest prevalence of CE [4]. However, the epidemiological situation in northern Tanzania has received little attention, and the only community-based survey on human CE in this region covered only one village at the border with Kenya and dates back to 1989 [17, 18].

In our study, we showed that *E. granulosus s.l*. continues to circulate in Maasai communities of this area, as demonstrated by the high percentage of infected livestock in both of the districts examined and the diagnosis of active CE cysts in young people, including a 7-year-old child. The detection and distribution of both active and inactive cysts in the screened population are in line with the results of US-based population studies carried out in other endemic areas of the world and, as expected in a chronic infection, acquired through exposure over time to a contaminated environment [16, 30, 31].

In the present study, CE cysts in humans were diagnosed only in villages in Ngorongoro District, with no CE diagnosed in participants from villages in Longido District, despite the parasite affecting a high percentage of slaughtered ruminants in the latter district. These data cannot be explained by a different origin of the animals slaughtered in the abattoirs of Longido District, since slaughtering of locally sourced animals is carried out in both districts. It is important to note that there are 33 villages in Longido District and 40 villages in Ngorongoro District. Therefore, our small sample of five villages across these two large districts in this pilot study prevents direct comparison of human CE prevalence between districts or statistical analysis to try to explain differences in prevalence observed between villages. However, our results do reveal some interesting features and suggest the presence of high environmental contamination by taeniid eggs in some villages that warrant further investigation. In particular, cases of CE were only detected in two very remote villages of Ngorongoro in which traditional forms of pastoralism were observed to predominate. By contrast, Enguserosambu in Ngorongoro District, in which no cases were detected, is known to have a mixture of livestock-based livelihoods, including pastoral, agro-pastoral and small holder-based systems [27]. Pastoral-based livelihoods continue to predominate in the two villages sampled in Longido District [27]. However, both of these villages are located on the main road between the large city of Arusha and the Kenyan border. These differences in remoteness and in livestock production systems may influence factors such as education, hygiene and cultural practices, and these in turn may influence the risk of human CE. *E. granulosus s.l.* cysts were detected in livestock in both districts, but the proportion of this parasite among genotyped cysts in slaughtered livestock was significantly lower in Longido District than in Ngorongoro District. This result suggests that there may be specific risk factors associated with animal husbandry and slaughtering practices in Ngorongoro District that differ from those in Longido District. It may also indicate a higher risk for CE among people in Ngorongoro District, as suggested and supported by the results of our US survey. Indeed, previous studies in Tanzania and in other endemic countries [17, 31] indicate that a complex, likely site-specific, combination of factors account for the overall risk of human infection. These include contamination with eggs, environmental conditions, animal husbandry and slaughtering practices, role of dogs and interaction with them, food and water sources and hygiene practices. The results of previous studies in northern Tanzania suggest a high level of environmental contamination (4–366 eggs/g soil and 10/100 eggs/ml water) with taeniid eggs [17], and a high prevalence (6–50%) of domestic dog infection [14, 17]. However, these studies did not apply diagnostic methods able to reliably identify the presence of *E. granulosus s.l.* and differentiate them from other taeniid parasites. Further investigation is needed to better map and estimate the prevalence of CE in these districts, to assess infection in dogs, to determine the distribution and level of environmental contamination with eggs and to identify potential risk factors and practices specific to these areas that could be the target for control of *E. granulosus* transmission in endemic villages in northern Tanzania. This would be particularly relevant given that cultural factors may be a less of a barrier if some of these practices have already been adopted in other Maasai communities.

*Echinococcus granulosus s.s.* (G1–G3) was the predominant species identified in all three livestock species evaluated in the abattoirs, and the only species identified from the CE cysts removed surgically from humans. This result is in line with the literature, which reports *E. granulosus s.s.* as responsible for the vast majority of infections worldwide in cases in which identification was achieved at the genotype level [32–34]. *E. granulosus s.s.* cysts were detected in both cattle and small ruminants and, although we did not carry out an analysis of cyst fertility in the different host species, it is possible that both types of livestock might be contributing to the perpetuation of the parasite life-cycle in the area [3, 35]. Other genotypes were also detected in livestock species, suggesting a complex epidemiology with a potential wider range of hosts involved. The potential epidemiological implications of this warrant further attention in this area of wildlife–livestock–human interaction where two-way transmission between domestic and sylvatic cycles may occur [12].

This study has several limitations. First, the number of villages per district was small, and the overall sample size of both humans and animals investigated was relatively small (due to the nature of the investigation, which is to be considered exploratory). Secondly, the sampling strategy was non-random (voluntary participation of humans and inspection of consecutive animals at abattoirs, applied because of ethical and logistical necessities, respectively). Third, we were unable to reach the target sample sizes and to assign a species to all cysts affecting slaughtered animals (due to financial constraints and COVID-19 pandemic-related problems in project investigators accessing the study sites to perform further US screening and inspect cysts at abattoir). Nonetheless, we were able demonstrate that multiple genotypes of *E. granulosus s.l.* continue to circulate in livestock in Maasai communities and pose an on-going threat to human health, with some Maasai communities appearing to be at greater risk than others. Public health strategies targeting transmission routes common to both *E. granulosus* and *T. multiceps*, such as interventions directed at the definitive host (e.g. regular treatment of domestic dogs with praziquantel) and/or intermediate hosts (e.g. application of safe slaughter of livestock and organ disposal practices), should be effective measures by which to control zoonotic and non-zoonotic cestode infections, with benefits for both humans and livestock. The widespread concern about the impact of *T. multiceps* on food security and livelihoods in pastoral communities and the positive response to on-going dissemination of education material on behavioural changes for the prevention of *T. multiceps* transmission (S. Cleaveland, personal communication) create important opportunities for linked control measures. Sheep vaccination also offers further opportunities for linked interventions. The EG95 vaccine against *E. granulosus* has been extensively tested and found to have a very high efficacy to control infection in small ruminants in New Zealand, Australia, Argentina, Chile, Iran, Romania and Morocco. This vaccine is now commercially available in China, Argentina and Morocco [36]. Although animal vaccination would lower the risks of human CE, the lack of appreciable economic benefits to farmers from the use of the vaccine may limit its adoption in low-income settings. The development of a vaccine for coenurosis [37, 38] provides further opportunities for linked small ruminant vaccination programmes that would be of direct and immediate benefit to pastoral communities in targeting both *E. granulosus* and *T. multiceps*.

## Conclusions

Multiple genotypes of *E. granulosus s.l.* are circulating in Maasai communities of northern Tanzania. Although the limitations of the present study prevent us from deriving precise estimates of infection prevalence across the region, our findings suggest that there has not been a major increase in the prevalence of human CE in Maasai communities over the past four decades: the apparent prevalence in the two villages in which we detected cases in Ngorongoro District (1–2.4%) are similar to those recorded in the late 1980s (1–1.4%). This stability contrasts with an apparent upsurge in cases of cerebral coenurosis in sheep and goats that has been reported in the same communities [19]. Several caveats remain, however, as both studies were both limited in size, and the infection prevalences of *T. multiceps* in livestock and CE in humans were determined using different approaches. Furthermore, the magnitude of a potential significant increase in incidence of *E. granulosus s.l.* might only become apparent in the years to come due to the long latency of symptoms in humans. Therefore, further surveys are clearly warranted to assess and monitor trends in CE across a larger proportion of high-risk pastoral communities. Nonetheless, regardless of infection trends, the detection of echinococcal cysts in livestock and human CE indicates that interventions targeting transmission routes common to both *E. granulosus s.l.* and *T. multiceps*, including deworming of dogs and effective disposal of infected tissues during livestock slaughter, would have dual benefits for preventing both human and livestock disease.

## Data Availability

The data file is available at http://dx.doi.org/10.5525/gla.researchdata.1299.

## Abbreviations

CE: Cystic echinococcosis
CI: Confidence interval
*COX1*: Cytochrome oxidase I gene
RFLP: Restriction fragment length polymorphism
US: Ultrasound
WHO-IWGE: WHO–Informal Working Group on Echinococcosis
